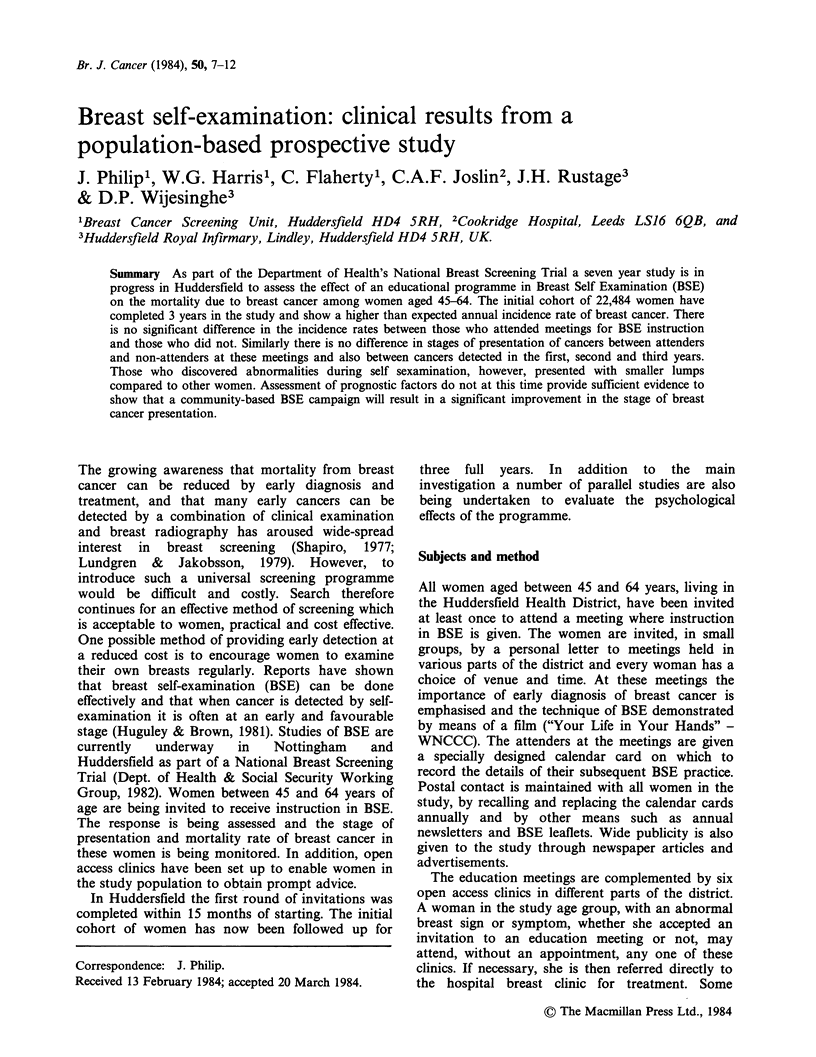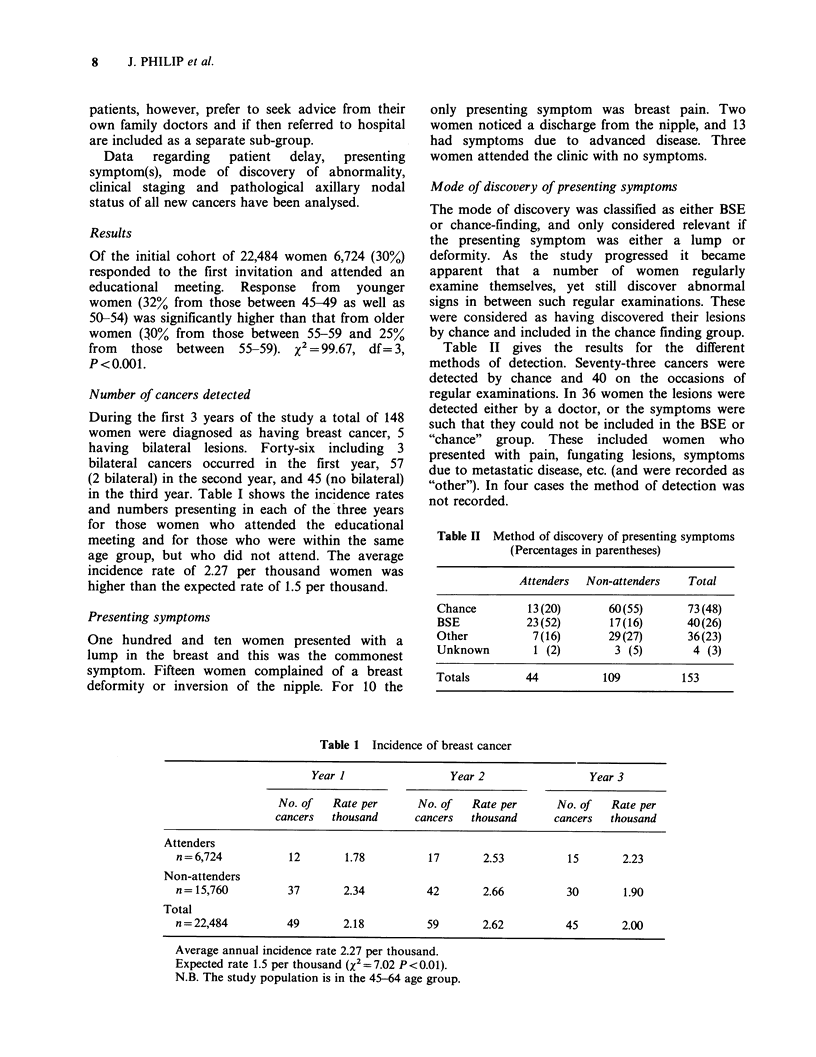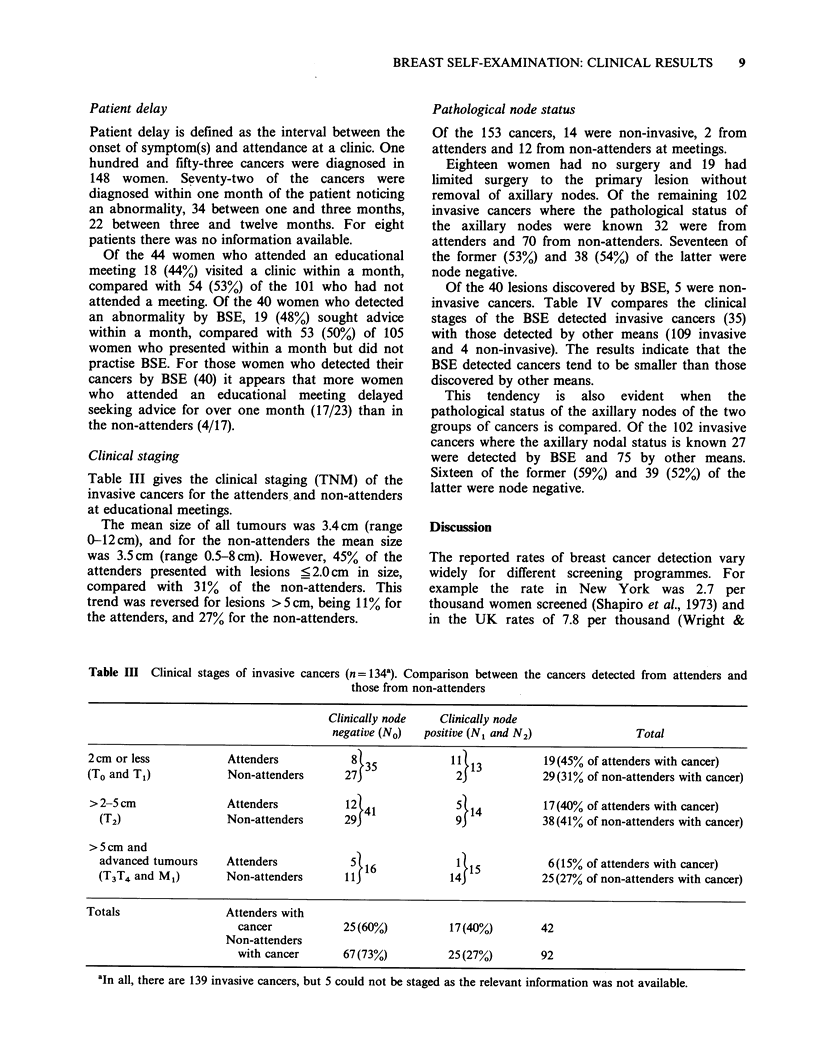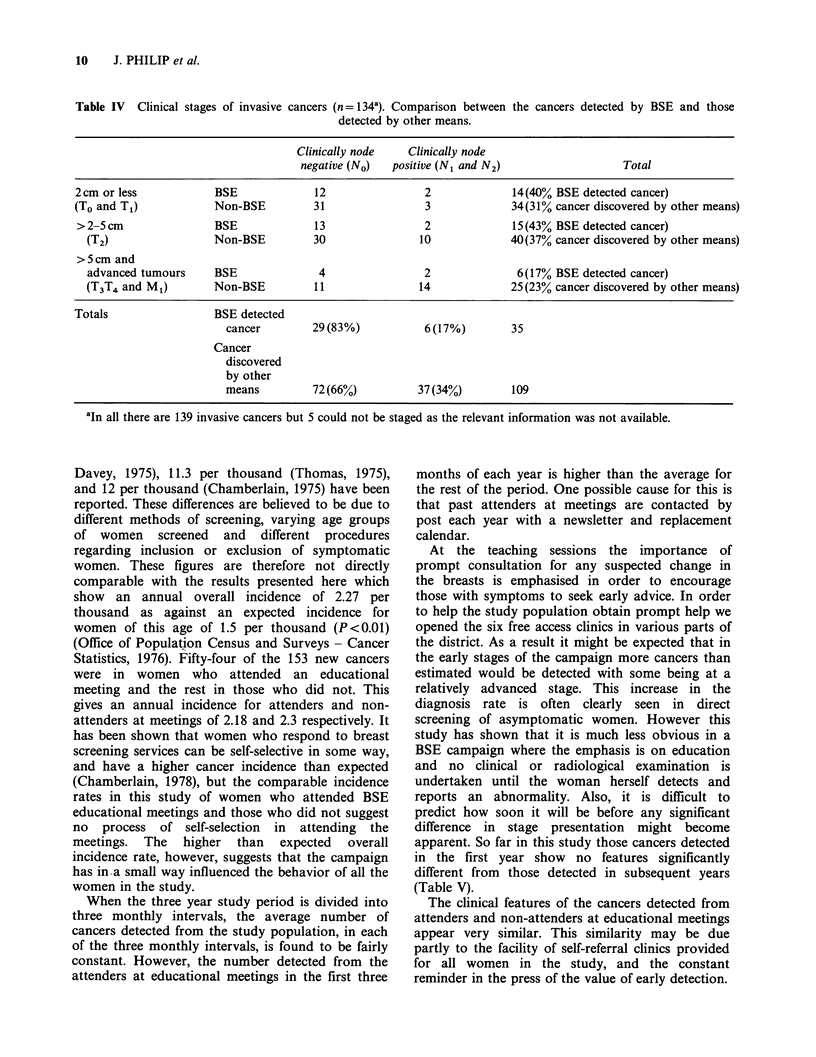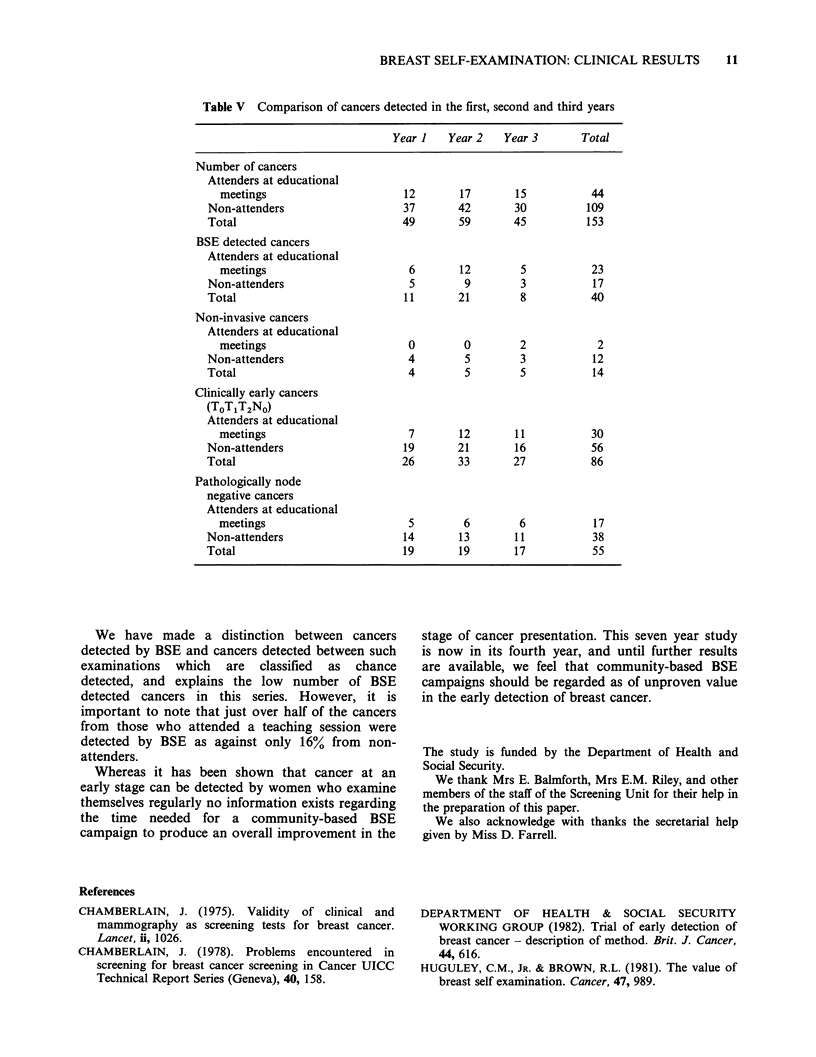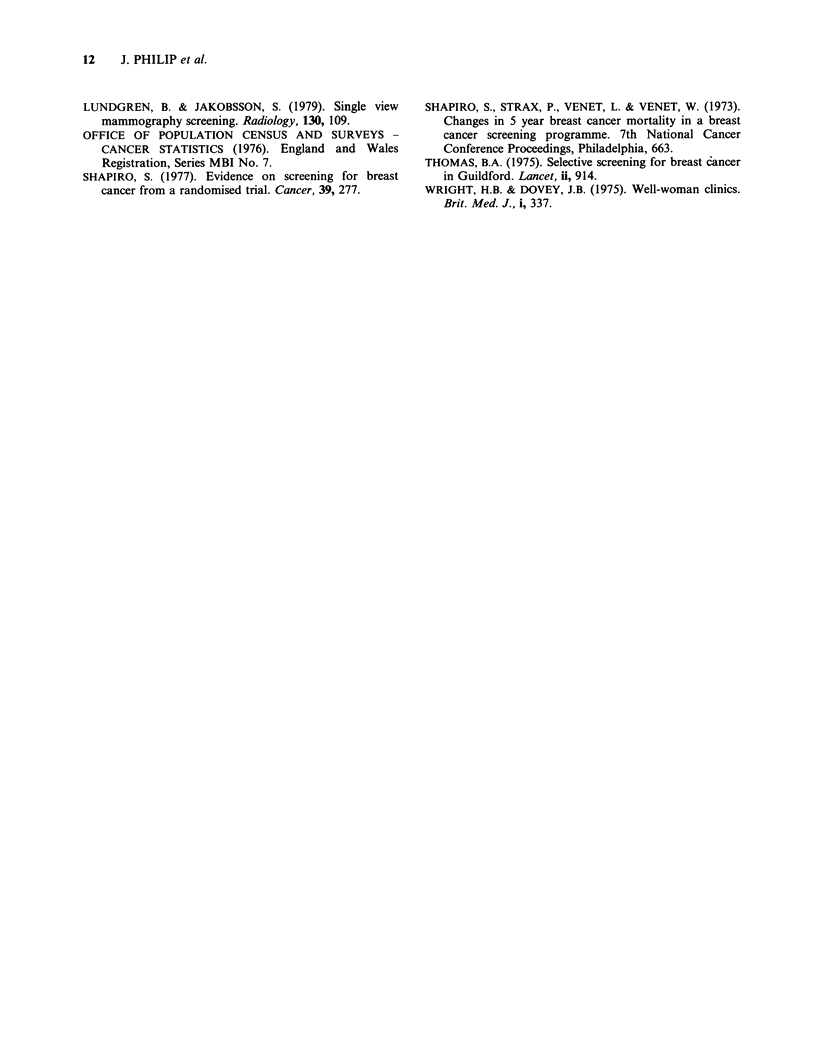# Breast self-examination: clinical results from a population-based prospective study.

**DOI:** 10.1038/bjc.1984.133

**Published:** 1984-07

**Authors:** J. Philip, W. G. Harris, C. Flaherty, C. A. Joslin, J. H. Rustage, D. P. Wijesinghe

## Abstract

As part of the Department of Health's National Breast Screening Trial a seven year study is in progress in Huddersfield to assess the effect of an educational programme in Breast Self Examination (BSE) on the mortality due to breast cancer among women aged 45-64. The initial cohort of 22,484 women have completed 3 years in the study and show a higher than expected annual incidence rate of breast cancer. There is no significant difference in the incidence rates between those who attended meetings for BSE instruction and those who did not. Similarly there is no difference in stages of presentation of cancers between attenders and non-attenders at these meetings and also between cancers detected in the first, second and third years. Those who discovered abnormalities during self examination, however, presented with smaller lumps compared to other women. Assessment of prognostic factors do not at this time provide sufficient evidence to show that a community-base BSE campaign will result in a significant improvement in the stage of breast cancer presentation.


*Br. J. Cancer* (1984), **50**, 7–12

### Breast self-examination: clinical results from a population-based prospective study

J. Philip[1], W.G. Harris[1], C. Flaherty[1], C.A.F. Joslin[2], J.H. Rustage[3] & D.P. Wijesinghe[3]

[1]*Breast Cancer Screening Unit, Huddersfield HD4 5RH,* [2]*Cookridge Hospital, Leeds LS16 6QB, and* [3]*Huddersfield Royal Infirmary, Lindley, Huddersfield HD4 5RH, UK.*

**Summary** As part of the Department of Health's National Breast Screening Trial a seven year study is in progress in Huddersfield to assess the effect of an educational programme in Breast Self Examination (BSE) on the mortality due to breast cancer among women aged 45–64. The initial cohort of 22,484 women have completed 3 years in the study and show a higher than expected annual incidence rate of breast cancer. There is no significant difference in the incidence rates between those who attended meetings for BSE instruction and those who did not. Similarly there is no difference in stages of presentation of cancers between attenders and non-attenders at these meetings and also between cancers detected in the first, second and third years. Those who discovered abnormalities during self sexamination, however, presented with smaller lumps compared to other women. Assessment of prognostic factors do not at this time provide sufficient evidence to show that a community-based BSE campaign will result in a significant improvement in the stage of breast cancer presentation.

The growing awareness that mortality from breast cancer can be reduced by early diagnosis and treatment, and that many early cancers can be detected by a combination of clinical examination and breast radiography has aroused wide-spread interest in breast screening (Shapiro, 1977; Lundgren & Jakobsson, 1979). However, to introduce such a universal screening programme would be difficult and costly. Search therefore continues for an effective method of screening which is acceptable to women, practical and cost effective. One possible method of providing early detection at a reduced cost is to encourage women to examine their own breasts regularly. Reports have shown that breast self-examination (BSE) can be done effectively and that when cancer is detected by self-examination it is often at an early and favourable stage (Huguley & Brown, 1981). Studies of BSE are currently underway in Nottingham and Huddersfield as part of a National Breast Screening Trial (Dept. of Health & Social Security Working Group, 1982). Women between 45 and 64 years of age are being invited to receive instruction in BSE. The response is being assessed and the stage of presentation and mortality rate of breast cancer in these women is being monitored. In addition, open access clinics have been set up to enable women in the study population to obtain prompt advice.

In Huddersfield the first round of invitations was completed within 15 months of starting. The initial cohort of women has now been followed up for three full years. In addition to the main investigation a number of parallel studies are also being undertaken to evaluate the psychological effects of the programme.

### Subjects and method

All women aged between 45 and 64 years, living in the Huddersfield Health District, have been invited at least once to attend a meeting where instruction in BSE is given. The women are invited, in small groups, by a personal letter to meetings held in various parts of the district and every woman has a choice of venue and time. At these meetings the importance of early diagnosis of breast cancer is emphasised and the technique of BSE demonstrated by means of a film ("Your Life in Your Hands" – WNCCC). The attenders at the meetings are given a specially designed calendar card on which to record the details of their subsequent BSE practice. Postal contact is maintained with all women in the study, by recalling and replacing the calendar cards annually and by other means such as annual newsletters and BSE leaflets. Wide publicity is also given to the study through newspaper articles and advertisements.

The education meetings are complemented by six open access clinics in different parts of the district. A woman in the study age group, with an abnormal breast sign or symptom, whether she accepted an invitation to an education meeting or not, may attend, without an appointment, any one of these clinics. If necessary, she is then referred directly to the hospital breast clinic for treatment. Some

Correspondence: J. Philip.
Received 13 February 1984; accepted 20 March 1984.

© The Macmillan Press Ltd., 1984

patients, however, prefer to seek advice from their own family doctors and if then referred to hospital are included as a separate sub-group.

Data regarding patient delay, presenting symptom(s), mode of discovery of abnormality, clinical staging and pathological axillary nodal status of all new cancers have been analysed.

### *Results*

Of the initial cohort of 22,484 women 6,724 (30%) responded to the first invitation and attended an educational meeting. Response from younger women (32% from those between 45–49 as well as 50–54) was significantly higher than that from older women (30% from those between 55–59 and 25% from those between 55–59). $\chi^2=99.67$, df=3, $P<0.001$.

### *Number of cancers detected*

During the first 3 years of the study a total of 148 women were diagnosed as having breast cancer, 5 having bilateral lesions. Forty-six including 3 bilateral cancers occurred in the first year, 57 (2 bilateral) in the second year, and 45 (no bilateral) in the third year. Table I shows the incidence rates and numbers presenting in each of the three years for those women who attended the educational meeting and for those who were within the same age group, but who did not attend. The average incidence rate of 2.27 per thousand women was higher than the expected rate of 1.5 per thousand.

### *Presenting symptoms*

One hundred and ten women presented with a lump in the breast and this was the commonest symptom. Fifteen women complained of a breast deformity or inversion of the nipple. For 10 the only presenting symptom was breast pain. Two women noticed a discharge from the nipple, and 13 had symptoms due to advanced disease. Three women attended the clinic with no symptoms.

### *Mode of discovery of presenting symptoms*

The mode of discovery was classified as either BSE or chance-finding, and only considered relevant if the presenting symptom was either a lump or deformity. As the study progressed it became apparent that a number of women regularly examine themselves, yet still discover abnormal signs in between such regular examinations. These were considered as having discovered their lesions by chance and included in the chance finding group.

Table II gives the results for the different methods of detection. Seventy-three cancers were detected by chance and 40 on the occasions of regular examinations. In 36 women the lesions were detected either by a doctor, or the symptoms were such that they could not be included in the BSE or "chance" group. These included women who presented with pain, fungating lesions, symptoms due to metastatic disease, etc. (and were recorded as "other"). In four cases the method of detection was not recorded.

**Table II** Method of discovery of presenting symptoms (Percentages in parentheses)

| | *Attenders* | *Non-attenders* | *Total* |
|---|---|---|---|
| Chance | 13 (20) | 60 (55) | 73 (48) |
| BSE | 23 (52) | 17 (16) | 40 (26) |
| Other | 7 (16) | 29 (27) | 36 (23) |
| Unknown | 1 (2) | 3 (5) | 4 (3) |
| Totals | 44 | 109 | 153 |

**Table 1** Incidence of breast cancer

| | *Year 1* | | *Year 2* | | *Year 3* | |
|---|---|---|---|---|---|---|
| | *No. of cancers* | *Rate per thousand* | *No. of cancers* | *Rate per thousand* | *No. of cancers* | *Rate per thousand* |
| Attenders $n=6{,}724$ | 12 | 1.78 | 17 | 2.53 | 15 | 2.23 |
| Non-attenders $n=15{,}760$ | 37 | 2.34 | 42 | 2.66 | 30 | 1.90 |
| Total $n=22{,}484$ | 49 | 2.18 | 59 | 2.62 | 45 | 2.00 |

Average annual incidence rate 2.27 per thousand.
Expected rate 1.5 per thousand ($\chi^2=7.02$ $P<0.01$).
N.B. The study population is in the 45–64 age group.

*Patient delay*

Patient delay is defined as the interval between the onset of symptom(s) and attendance at a clinic. One hundred and fifty-three cancers were diagnosed in 148 women. Seventy-two of the cancers were diagnosed within one month of the patient noticing an abnormality, 34 between one and three months, 22 between three and twelve months. For eight patients there was no information available.

Of the 44 women who attended an educational meeting 18 (44%) visited a clinic within a month, compared with 54 (53%) of the 101 who had not attended a meeting. Of the 40 women who detected an abnormality by BSE, 19 (48%) sought advice within a month, compared with 53 (50%) of 105 women who presented within a month but did not practise BSE. For those women who detected their cancers by BSE (40) it appears that more women who attended an educational meeting delayed seeking advice for over one month (17/23) than in the non-attenders (4/17).

*Clinical staging*

Table III gives the clinical staging (TNM) of the invasive cancers for the attenders and non-attenders at educational meetings.

The mean size of all tumours was 3.4 cm (range 0–12 cm), and for the non-attenders the mean size was 3.5 cm (range 0.5–8 cm). However, 45% of the attenders presented with lesions ≤2.0 cm in size, compared with 31% of the non-attenders. This trend was reversed for lesions >5 cm, being 11% for the attenders, and 27% for the non-attenders.

*Pathological node status*

Of the 153 cancers, 14 were non-invasive, 2 from attenders and 12 from non-attenders at meetings.

Eighteen women had no surgery and 19 had limited surgery to the primary lesion without removal of axillary nodes. Of the remaining 102 invasive cancers where the pathological status of the axillary nodes were known 32 were from attenders and 70 from non-attenders. Seventeen of the former (53%) and 38 (54%) of the latter were node negative.

Of the 40 lesions discovered by BSE, 5 were non-invasive cancers. Table IV compares the clinical stages of the BSE detected invasive cancers (35) with those detected by other means (109 invasive and 4 non-invasive). The results indicate that the BSE detected cancers tend to be smaller than those discovered by other means.

This tendency is also evident when the pathological status of the axillary nodes of the two groups of cancers is compared. Of the 102 invasive cancers where the axillary nodal status is known 27 were detected by BSE and 75 by other means. Sixteen of the former (59%) and 39 (52%) of the latter were node negative.

### Discussion

The reported rates of breast cancer detection vary widely for different screening programmes. For example the rate in New York was 2.7 per thousand women screened (Shapiro *et al.*, 1973) and in the UK rates of 7.8 per thousand (Wright &

**Table III** Clinical stages of invasive cancers ($n=134^a$). Comparison between the cancers detected from attenders and those from non-attenders

| | | *Clinically node negative* ($N_0$) | *Clinically node positive* ($N_1$ *and* $N_2$) | *Total* |
|---|---|---|---|---|
| 2 cm or less ($T_0$ and $T_1$) | Attenders | 8 } 35 | 11 } 13 | 19 (45% of attenders with cancer) |
| | Non-attenders | 27 | 2 | 29 (31% of non-attenders with cancer) |
| >2–5 cm ($T_2$) | Attenders | 12 } 41 | 5 } 14 | 17 (40% of attenders with cancer) |
| | Non-attenders | 29 | 9 | 38 (41% of non-attenders with cancer) |
| >5 cm and advanced tumours ($T_3T_4$ and $M_1$) | Attenders | 5 } 16 | 1 } 15 | 6 (15% of attenders with cancer) |
| | Non-attenders | 11 | 14 | 25 (27% of non-attenders with cancer) |
| Totals | Attenders with cancer | 25 (60%) | 17 (40%) | 42 |
| | Non-attenders with cancer | 67 (73%) | 25 (27%) | 92 |

[a]In all, there are 139 invasive cancers, but 5 could not be staged as the relevant information was not available.

**Table IV** Clinical stages of invasive cancers ($n=134^a$). Comparison between the cancers detected by BSE and those detected by other means.

| | | *Clinically node negative* ($N_0$) | *Clinically node positive* ($N_1$ *and* $N_2$) | *Total* |
|---|---|---|---|---|
| 2 cm or less ($T_0$ and $T_1$) | BSE | 12 | 2 | 14 (40% BSE detected cancer) |
| | Non-BSE | 31 | 3 | 34 (31% cancer discovered by other means) |
| >2–5 cm ($T_2$) | BSE | 13 | 2 | 15 (43% BSE detected cancer) |
| | Non-BSE | 30 | 10 | 40 (37% cancer discovered by other means) |
| >5 cm and advanced tumours ($T_3T_4$ and $M_1$) | BSE | 4 | 2 | 6 (17% BSE detected cancer) |
| | Non-BSE | 11 | 14 | 25 (23% cancer discovered by other means) |
| Totals | BSE detected cancer | 29 (83%) | 6 (17%) | 35 |
| | Cancer discovered by other means | 72 (66%) | 37 (34%) | 109 |

[a]In all there are 139 invasive cancers but 5 could not be staged as the relevant information was not available.

Davey, 1975), 11.3 per thousand (Thomas, 1975), and 12 per thousand (Chamberlain, 1975) have been reported. These differences are believed to be due to different methods of screening, varying age groups of women screened and different procedures regarding inclusion or exclusion of symptomatic women. These figures are therefore not directly comparable with the results presented here which show an annual overall incidence of 2.27 per thousand as against an expected incidence for women of this age of 1.5 per thousand ($P<0.01$) (Office of Population Census and Surveys – Cancer Statistics, 1976). Fifty-four of the 153 new cancers were in women who attended an educational meeting and the rest in those who did not. This gives an annual incidence for attenders and non-attenders at meetings of 2.18 and 2.3 respectively. It has been shown that women who respond to breast screening services can be self-selective in some way, and have a higher cancer incidence than expected (Chamberlain, 1978), but the comparable incidence rates in this study of women who attended BSE educational meetings and those who did not suggest no process of self-selection in attending the meetings. The higher than expected overall incidence rate, however, suggests that the campaign has in a small way influenced the behavior of all the women in the study.

When the three year study period is divided into three monthly intervals, the average number of cancers detected from the study population, in each of the three monthly intervals, is found to be fairly constant. However, the number detected from the attenders at educational meetings in the first three months of each year is higher than the average for the rest of the period. One possible cause for this is that past attenders at meetings are contacted by post each year with a newsletter and replacement calendar.

At the teaching sessions the importance of prompt consultation for any suspected change in the breasts is emphasised in order to encourage those with symptoms to seek early advice. In order to help the study population obtain prompt help we opened the six free access clinics in various parts of the district. As a result it might be expected that in the early stages of the campaign more cancers than estimated would be detected with some being at a relatively advanced stage. This increase in the diagnosis rate is often clearly seen in direct screening of asymptomatic women. However this study has shown that it is much less obvious in a BSE campaign where the emphasis is on education and no clinical or radiological examination is undertaken until the woman herself detects and reports an abnormality. Also, it is difficult to predict how soon it will be before any significant difference in stage presentation might become apparent. So far in this study those cancers detected in the first year show no features significantly different from those detected in subsequent years (Table V).

The clinical features of the cancers detected from attenders and non-attenders at educational meetings appear very similar. This similarity may be due partly to the facility of self-referral clinics provided for all women in the study, and the constant reminder in the press of the value of early detection.

**Table V** Comparison of cancers detected in the first, second and third years

| | *Year 1* | *Year 2* | *Year 3* | *Total* |
|---|---|---|---|---|
| Number of cancers | | | | |
| Attenders at educational meetings | 12 | 17 | 15 | 44 |
| Non-attenders | 37 | 42 | 30 | 109 |
| Total | 49 | 59 | 45 | 153 |
| BSE detected cancers | | | | |
| Attenders at educational meetings | 6 | 12 | 5 | 23 |
| Non-attenders | 5 | 9 | 3 | 17 |
| Total | 11 | 21 | 8 | 40 |
| Non-invasive cancers | | | | |
| Attenders at educational meetings | 0 | 0 | 2 | 2 |
| Non-attenders | 4 | 5 | 3 | 12 |
| Total | 4 | 5 | 5 | 14 |
| Clinically early cancers ($T_0T_1T_2N_0$) | | | | |
| Attenders at educational meetings | 7 | 12 | 11 | 30 |
| Non-attenders | 19 | 21 | 16 | 56 |
| Total | 26 | 33 | 27 | 86 |
| Pathologically node negative cancers | | | | |
| Attenders at educational meetings | 5 | 6 | 6 | 17 |
| Non-attenders | 14 | 13 | 11 | 38 |
| Total | 19 | 19 | 17 | 55 |

We have made a distinction between cancers detected by BSE and cancers detected between such examinations which are classified as chance detected, and explains the low number of BSE detected cancers in this series. However, it is important to note that just over half of the cancers from those who attended a teaching session were detected by BSE as against only 16% from non-attenders.

Whereas it has been shown that cancer at an early stage can be detected by women who examine themselves regularly no information exists regarding the time needed for a community-based BSE campaign to produce an overall improvement in the stage of cancer presentation. This seven year study is now in its fourth year, and until further results are available, we feel that community-based BSE campaigns should be regarded as of unproven value in the early detection of breast cancer.

The study is funded by the Department of Health and Social Security.

We thank Mrs E. Balmforth, Mrs E.M. Riley, and other members of the staff of the Screening Unit for their help in the preparation of this paper.

We also acknowledge with thanks the secretarial help given by Miss D. Farrell.

**References**

CHAMBERLAIN, J. (1975). Validity of clinical and mammography as screening tests for breast cancer. *Lancet*, **ii**, 1026.

CHAMBERLAIN, J. (1978). Problems encountered in screening for breast cancer screening in Cancer UICC Technical Report Series (Geneva), **40**, 158.

DEPARTMENT OF HEALTH & SOCIAL SECURITY WORKING GROUP (1982). Trial of early detection of breast cancer – description of method. *Brit. J. Cancer*, **44**, 616.

HUGULEY, C.M., JR. & BROWN, R.L. (1981). The value of breast self examination. *Cancer*, **47**, 989.

LUNDGREN, B. & JAKOBSSON, S. (1979). Single view mammography screening. *Radiology,* **130,** 109.

OFFICE OF POPULATION CENSUS AND SURVEYS – CANCER STATISTICS (1976). England and Wales Registration, Series MBI No. 7.

SHAPIRO, S. (1977). Evidence on screening for breast cancer from a randomised trial. *Cancer,* **39,** 277.

SHAPIRO, S., STRAX, P., VENET, L. & VENET, W. (1973). Changes in 5 year breast cancer mortality in a breast cancer screening programme. 7th National Cancer Conference Proceedings, Philadelphia, 663.

THOMAS, B.A. (1975). Selective screening for breast cancer in Guildford. *Lancet,* **ii,** 914.

WRIGHT, H.B. & DOVEY, J.B. (1975). Well-woman clinics. *Brit. Med. J.,* **i,** 337.